# FoxP3 and Bcl-xL cooperatively promote regulatory T cell persistence and prevention of arthritis development

**DOI:** 10.1186/ar2983

**Published:** 2010-04-12

**Authors:** Rizwanul Haque, Fengyang Lei, Xiaofang Xiong, Yuzhang Wu, Jianxun Song

**Affiliations:** 1 Department of Microbiology & Immunology and Penn State Hershey Cancer Institute, The Pennsylvania State University College of Medicine, 500 University Drive, Hershey, PA 17033, USA; 2 Institute of Immunology, The Third Military Medical University, 30 Gaotanyan Street, Chongqing 400038, PR China

## Abstract

**Introduction:**

Forkhead box p3 (FoxP3)-expressing regulatory T cells (Tregs) have been clearly implicated in the control of autoimmune disease in murine models. In addition, ectopic expression of FoxP3 conveys a Treg phenotype to CD4^+ ^T cells, lending itself to therapeutic use in the prevention of rheumatoid arthritis (RA). In this study, we generated therapeutically active Tregs with an increased life span and hence greater therapeutic potential.

**Methods:**

We used retrovirus-mediated transduction to introduce FoxP3 or FoxP3 with anti-apoptotic Bcl-2 family molecule Bcl-xL linked by a 2A picornavirus self-cleaving peptide into CD4^+ ^T cells to generate Tregs. In addition, by using *in vitro *functional analyses and adoptive immunotherapy in a murine model of RA, we demonstrated that these Tregs were highly reactive.

**Results:**

We found that CD4^+ ^T cells expressing both FoxP3 and Bcl-xL were able to differentiate into functional Tregs, which have a long-term survival advantage over cells transduced with FoxP3 alone. In an *in vivo *murine model, adoptive transfer of Tregs expressing both FoxP3 and Bcl-xL demonstrated more effective suppression of RA than CD4^+ ^T cells expressing FoxP3 alone.

**Conclusions:**

FoxP3 and Bcl-xL can cooperatively promote the differentiation and persistence of Tregs, with the capacity to prevent arthritis. Our results provide a novel approach for generating highly reactive Tregs for augmenting cellular immunotherapy for autoimmune disease.

## Introduction

Regulatory T cells (Tregs) are a specialized subpopulation of T cells that act to suppress activation of the immune system and thereby maintain immune system homeostasis and tolerance to self-antigens. Tregs are defined by expression of the forkhead family transcription factor FoxP3 (forkhead box p3), and CD4^+^CD25^+^FoxP3^+ ^Tregs are referred to as 'naturally occurring' Tregs [1]. Tregs comprise about 5% to 10% of the mature CD4^+ ^helper T-cell subpopulation in mice and about 1% to 2% of CD4^+ ^T cells in humans. It has been shown that functional Tregs can be generated from naive CD4^+ ^T cells by gene transduction of FoxP3 [2-4]. The presence of transforming growth factor-beta 1 (TGF-β1), interleukin-10 (IL-10), and IL-35 is also required for maximal suppressive activity of Tregs [5-7]. However, the mechanisms by which Tregs exert their suppressor/regulatory activity have not been fully characterized and are the subject of intensive research.

T-cell receptor (TCR) engagement or co-stimulatory signals (for example, CD28) or both lead to expression of several Bcl-2 family members, including Bcl-xL, Bcl-2, and Bfl-1, which control T-cell survival [8,9]. In addition, these signals modulate expression of FoxP3, which controls differentiation of Tregs [10-13]. Previously, we demonstrated that retrovirus-mediated transduction of target genes of co-stimulation (for example, Bcl-xL, IKKβ [inhibitor of kappaB kinase beta], survivin, and aurora B) could promote T-cell functions [14-17]. Therefore, we hypothesize that gene transduction of naive CD4^+ ^T cells with FoxP3 and Bcl-xL can induce the generation of highly reactive Tregs, which may be used in the treatment of autoimmune disease.

Recent strategies have used the foot-and-mouth disease virus 2A or 2A-like elements to create multicistronic vectors capable of generating multiple proteins from the same transcript. We previously demonstrated that a single 2A peptide-linked retroviral vector can be used successfully to generate reliable and versatile gene therapy vectors that can be used in biomedical research [18]. To understand whether FoxP3 and Bcl-xL can cooperatively regulate differentiation and survival of Tregs, we used retrovirus-mediated transduction to introduce FoxP3 and Bcl-xL linked by a 2A peptide into naive CD4^+ ^T cells. We found that co-expression of FoxP3 and Bcl-xL in CD4^+ ^cells is critical for augmenting the differentiation and persistence of Tregs. Most significantly, the co-introduction of these molecules into CD4^+ ^T cells resulted in their ability to significantly block the development of arthritis in a well-established murine model. Thus, these data indicate that FoxP3 and Bcl-xL can cooperatively promote differentiation and function of Tregs. Furthermore, genetic modification with FoxP3 and Bcl-xL using vectors containing the 2A sequence is able to generate highly reactive Tregs that could be used for augmented cellular immunotherapy for autoimmune disease.

## Materials and methods

### Mice

DAB/1J and C57BL/6J mice were purchased from The Jackson Laboratory (Bar Harbor, ME, USA). OT-II TCR-transgenic mice, expressing a TCR composed of variable (Vβ5 and Vα2) chains responsive to the I-A^b^-restricted ovalbumin (OVA) peptide 323-339 (ISQAVHAAHAEINAGR), were maintained by breeding with C57BL/6J mice. All experiments were in compliance with the regulations of the Pennsylvania State University College of Medicine Animal Care Committee and were in accordance with guidelines of the Association for the Assessment and Accreditation of Laboratory Animal Care.

### Antibodies

Anti-CD28 (37.51), mouse IL-2, and interferon-gamma (IFN-γ) were from BD Pharmingen (San Diego, CA, USA). Anti-actin (C2, sc-8432) for Western blot was purchased from Santa Cruz Biotechnology, Inc. (Santa Cruz, CA, USA). Anti-Bcl-xL (#2762), peroxidase-conjugated anti-rabbit (#7054), and anti-mouse Ig (#7056) for Western blot were purchased from Cell Signaling Technology (Beverly, MA, USA). All FITC (fluorescein isothiocyanate)-, PE (phycoerythrin)-, Cy5 (cyanine 5)-, and APC (antigen-presenting cell)-conjugated antibodies were purchased from BioLegend (San Diego, CA, USA).

### T cells

Naive CD4^+ ^T cells from spleen and lymph nodes of OT-II mice were enriched by magnetic sorting with the CD4^+ ^T Cell Isolation Kit (#130-090-860; Miltenyi Biotec Inc., Auburn, CA, USA). The cells were more than 90% CD4^+^, and more than 95% of these cells expressed a naive phenotype and the appropriate TCR as determined by flow cytometry at the Penn State Hershey flow cytometry core facility.

### T-cell cultures

T cells were cultured in 24-well plates containing 1 mL of RPMI 1640 (Invitrogen Corporation, Carlsbad, CA, USA) with 10% fetal calf serum (HyClone, Logan, UT, USA). Naive CD4^+ ^T cells were stimulated with plate-bounded anti-CD3 (2C11, 4 μg/mL) and soluble anti-CD28 (37.51, 4 μg/mL) antibodies.

### Retrovirus-mediated transduction

cDNA corresponding to human Bc1xL was subcloned into the murine bicistronic retroviral expression vector Mig [18]. Mig-FoxP3 was a gift from Alexander Y Rudensky (Department of Immunology, University of Washington, Seattle, WA, USA) [19]. Retrovirus-mediated transduction was performed as described previously [16]. Briefly, T cells (1 × 10^6^) were stimulated with plate-bounded anti-CD3 and soluble anti-CD28 antibodies. After 2 days, the supernatant was replaced with 1 mL of viral supernatant containing 5 μg/mL Polybrene (Sigma-Aldrich, St. Louis, MO, USA), and the cells were sedimented by centrifugation for 1.5 hours at 32°C and incubated at 32°C for 8 hours. This step was repeated the following day. Viral supernatant was removed and replaced with fresh medium, and T cells were re-cultured. Expression of green fluorescent protein (GFP) in CD4^+ ^T cells, indicative of retrovirus-mediated transduction, was determined by flow cytometry. GFP-expressing T cells were purified by cell sorting using a MoFlo high-performance cell sorter (Beckman Coulter, Fullerton, CA, USA).

### Collagen-induced arthritis

Male DBA/1J mice (greater than 4 months of age) were injected at the base of the tail with 0.1 mL of emulsion containing 100 μg of bovine type II collagen (CII) (Chondrex, Redmond, WA, USA) in complete Freund's adjuvant (CFA) (Chondrex) using a 1-mL glass tuberculin syringe with a 26-guage needle. Mice were assessed for clinical arthritis in the paws, with each paw individually scored using a 4-point scale: 0, normal paw; 1, minimal swelling or redness; 2, redness and swelling involving the entire forepaw; 3, redness and swelling involving the entire limb; 4, joint deformity or ankylosis or both. Animals achieving a clinical score of 4 were euthanized.

### Histological evaluation

Mice were sacrificed on day 60 after the challenge of CII. Hind foot paws were amputated, fixed in 10% formalin, and decalcified in Formical-4 (Decal Chemical Corporation, Tallman, NY, USA). The tissues were embedded in paraffin, sectioned at 4 μm, and stained with hematoxylin and eosin (HE) or Safranin O-fast green as described before [20]. HE staining for bone erosions was scored using a semiquantitative scoring system from 0 to 4 (0 = no erosions, 4 = extended erosions and destruction of bone). Safranin O staining for the loss of proteoglycans was scored with a semiquantitative scoring system (0 to 3), where 0 represents no loss of proteoglycans and 3 indicates complete loss of staining for proteoglycans.

### Antibody detection

Blood samples were collected from the orbital sinus or by heart puncture on day 60 after primary immunization with CII. Total IgG was measured using the Easy-Titer IgG Assay Kit (Pierce, Rockford, IL, USA) in accordance with the recommendations of the manufacturer. The levels of anti-CII IgG in these sera were measured by enzyme-linked immunosorbent assay (ELISA) as described before [21]. Briefly, a 96-well microplate (Nunc, Roskilde, Denmark) was coated with 5 μg/mL CII at 4°C overnight followed by blocking with 0.2% bovine serum albumin and 0.05% Tween-20 in phosphate-buffered saline (PBS) at 4°C for 6 hours. Test samples were appropriately diluted with PBS containing 2% normal goat serum and added at 200 μL/well. After storage at 4°C overnight, wells were washed three times with PBS. Next, 100 μL of 1:1,000-diluted rabbit anti-mouse IgG1 or IgG2a conjugated with biotin and 100 μL of streptavvidin-peroxidase secondary antibody (Rockland Immunochemicals Inc., Gilbertsville, PA, USA) was added to each well and incubated at 4°C for 2 hours and 1 hour, respectively. After thorough washing, bound peroxidase activity was assayed by incubating samples at room temperature with 100 μL of TMB (3,3',5,5' tetramethylbenzidine) substrate (BioLegend). Absorbance at 405 nm was measured using a microplate reader.

### Adoptive cell transfer

T cells were cultured with CD3/CD28 and transduced on day 2/3 with retroviral vectors as described previously [16]. Cells were recultured for 3 more days. GFP^+ ^CD4^+ ^T cells were sorted by a MoFlo high-performance cell sorter, and 2.5 × 10^6 ^cells were injected intravenously into the tail vein of male DBA/1J mice that had been induced to develop collagen-induced arthritis (CIA) 15 days prior by immunization with CII.

### Cytokine secretion, cell recovery, and proliferation

Cytokines were measured by ELISA [15]. T-cell survival *in vitro *was determined by trypan blue exclusion. Cell proliferation was measured by incorporation of 5-bromo-2-deoxyuridine (BrdU) (Invitrogen Corporation) overnight, staining with anti-BrdU, and analysis by flow cytometry. Alternatively, incorporation of [^3^H] thymidine (1 μCi/well; PerkinElmer Inc., Boston, MA, USA) during the last 12 hours of culture was measured.

### Immunoblotting

Live CD4^+ ^T cells were lysed in ice-cold RIPA lysis buffer (20 mM Tris-HCl [pH 7.5], 150 mM NaCl, 1 mM Na_2_EDTA, 1 mM EGTA, 1% Triton, 2.5 mM sodium pyrophosphate, 1 mM beta-glycerophosphate, 1 mM Na_3_VO_4_, and 1 μg/mL leupeptin) for 30 minutes. Insoluble material was removed by centrifugation, and lysates were used for Western blotting. Protein content was determined by a Bio-Rad protein assay kit (Bio-Rad Laboratories, Inc., Hercules, CA, USA). Equal amounts of protein (40 μg) were loaded onto 4% to 12% NuPage Bis-Tris precasting gels and separated by SDS-PAGE, transferred onto nitrocellulose membrane (Invitrogen Corporation), and immunoblotted. All blots were developed with the ECL (enhanced chemiluminescence) immunodetection system (Amersham Pharmacia Biotech, now part of GE Healthcare, Little Chalfont, Buckinghamshire, UK).

## Results

### Expression of FoxP3 and Bcl-xL using 2A gene sequence in primary CD4^+ ^T cells

**Figure 1:**
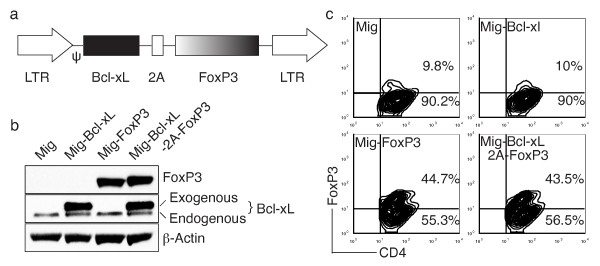
**Expression of FoxP3 and Bcl-xL using a 2A gene sequence in primary CD4^+ ^T cells**. **(a) **Schematic representation of the retrovirus construct expressing Bcl-xL and FoxP3. ψ, packaging signal; 2A, picornavirus self-cleaving 2A sequence. **(b) **Naive CD4^+ ^T cells from C57BL/6J mice were stimulated with anti-CD3 plus anti-CD28 antibodies. On day 2/3, T cells were transduced with retroviral vectors expressing green fluorescent protein (GFP) (Mig), GFP with FoxP3 (Mig-FoxP3), GFP with Bcl-xL (Mig-Bcl-xL), or GFP with Bcl-xL and FoxP3 (Mig-Bcl-xL-2A-FoxP3). On day 8 of primary culture, GFP^+ ^CD4^+ ^T cells were sorted, and protein expression of FoxP3, Bcl-xL, and β-actin was determined by Western blotting. Data are representative of three independent experiments. **(c) **Intracellular FoxP3 expression on day 6 of primary culture was analyzed by flow cytometry after gating on live CD4^+ ^T cells. Data are representative of three independent experiments.

Previously, we used the 2A peptide regions from picornavirus TaV (abbreviated herein as T2A) to generate multicistronic cassettes that linked Bcl-xL and survivin to make a single fragment encoding two proteins [18]. To generate reliable and versatile constructs to transduce primary CD4^+ ^T cells that permit expression of FoxP3 and Bcl-xL genes, we used the T2A peptide to generate a retroviral vector with efficient translation of two cistrons (for example, FoxP3 and Bcl-xL) (Figure 1a). The human Bcl-xL gene and T2A were excised from Mig-Bcl-xL-2A-survivin [18] and subcloned into Mig-FoxP3. Thus, Bcl-xL and FoxP3 were linked with the 2A sequence in the Mig vector. The integrity of the Mig-Bcl-xL-2A-FoxP3 construct was confirmed by DNA sequencing (data not shown), and protein expression was verified by Western blot (Figure 1b). Both Bcl-xL expression and FoxP3 expression were induced by retroviral infection of CD4^+ ^T cells following transduction with retroviral constructs expressing the individual genes or both genes (Figure 1b). By this method, approximately half of the cultured CD4^+ ^T cells were induced to express FoxP3 following retrovirus-mediated transduction as visualized by flow cytometry (Figure 1c).

### FoxP3 and Bcl-xL promote sustained survival of Tregs *in vitro*

**Figure 2:**
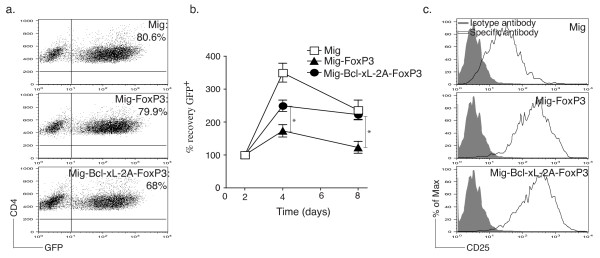
**Retrovirus-mediated transduction of FoxP3 and Bcl-xL promotes survival of regulatory T cells *in vitro***. Naive CD4^+ ^T cells from C57BL/6J mice were stimulated with anti-CD3 plus anti-CD28 antibodies, transduced on days 2/3 with retroviral vectors expressing green fluorescent protein (GFP), GFP with FoxP3, or GFP with Bcl-xL and FoxP3, and then recultured without any further stimulation. **(a) **Live CD4^+ ^GFP^+ ^T cells were visualized by flow cytometry on day 6 of culture. Data are representative of three independent experiments. **(b) **GFP^+ ^T-cell recovery was normalized to take into account differences in initial transduction efficiency between cultures. Numbers of GFP^+ ^cells present on day 2 were assigned a value of 100%, and numbers surviving on days 4, 6, and 8 were used to calculate the percentage recovery relative to day 2. Data represent the mean percentage change ± standard deviation from three separate experiments (*P <0.05, Student unpaired *t *test). **(c) **CD25 expression on day 4 of primary culture was analyzed by flow cytometry after gating on live GFP^+ ^CD4^+ ^T cells. Data are representative of three independent experiments.

To determine whether induced co-expression of FoxP3 and Bcl-xL promotes the persistence of Tregs, we transduced naive CD4^+ ^T cells with the GFP-IRES (GFP-internal ribosome entry site) retroviral vector containing FoxP3 and Bcl-xL (Mig-Bcl-xL-2A-FoxP3) (Figure 2a). After 2 to 3 days of transduction, the recovery of live T cells was monitored by GFP expression. On day 4 post-transduction, we found that the expansion of CD4^+ ^T cells that had been transduced with both FoxP3 and Bcl-xL was greater than the expansion of cells transduced with FoxP3 alone (Figure 2b). Longer-term culture over 8 days showed that co-expression of FoxP3 and Bcl-xL enhanced the ability of CD4^+ ^T cells to survive as compared with that of CD4^+ ^T cells transduced with FoxP3 alone (Figure 2b). This result was not due to differences in FoxP3 expression between cells transduced with both FoxP3 and Bcl-xL versus FoxP3 alone, indicating that exogenous Bcl-xL did not affect FoxP3 expression (Figure 1b). Moreover, expression of CD25, a marker for Tregs, was significantly upregulated by day 4 in the CD4^+ ^GFP^+ ^T cells that were transduced with FoxP3 or with FoxP3 plus Bcl-xL as compared with cells in the vector control group, suggesting that these cells were characteristic of Tregs after gene transfection (Figure 2c). Similar profiles were observed in the expression of cytotoxic T-lymphocyte-associated molecule-4 (CTLA-4) (data not shown). Thus, co-expression of FoxP3 and Bcl-xL induced from a single retroviral vector produced a Treg phenotype in the resulting cells and provided increased long-term survival of these cells.

### FoxP3 and Bcl-xL enhance the suppressive activity of Tregs *in vitro*

**Figure 3:**
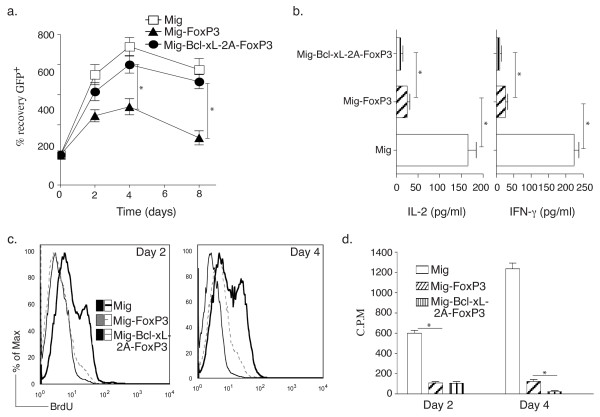
**Retrovirus-mediated transduction of FoxP3 and Bcl-xL enhances suppressive activity of regulatory T cells (Tregs) *in vitro***. Naive CD4^+ ^T cells from C57BL/6J mice were stimulated with anti-CD3 plus anti-CD28 antibodies (Abs). On day 2/3, T cells were transduced with retroviral vectors expressing green fluorescent protein (GFP), GFP with FoxP3, or GFP with Bcl-xL and FoxP3. On day 6 of primary culture, GFP^+ ^CD4^+ ^Tregs were sorted and re-stimulated with anti-CD3 and anti-CD28 Abs **(a) **or co-cultured with naive CD4^+ ^T cells from C57BL/6J mice (Tregs/T cells = 1:1) stimulated with anti-CD3 and anti-CD28 Abs for various periods **(b, c)**. (a) Recall survival of Tregs, based on recovery of GFP^+ ^CD4^+ ^T cells over time. Cell numbers present on day 0 were assigned a value of 100%, and cell numbers surviving on day 2 to day 8 were used to calculate the percentage recovery. Data represent the mean percentage change ± standard deviation from three separate experiments. (b) Interleukin-2 (IL-2) production and interferon-gamma (IFN-γ) production were measured by enzyme-linked immunosorbent assay at 48 hours. Data are representative of three independent experiments. Proliferation on day 2 or 4 was analyzed by flow cytometry for 5-bromo-2-deoxyuridine (BrdU) incorporation (c) and thymidine incorporation during the last 12 hours **(d)**. Data are representative of three experiments. *P <0.05, Student unpaired *t *test.

To investigate whether co-expression of FoxP3 and Bcl-xL promotes suppressive activity of Tregs, CD4^+ ^T cells expressing FoxP3 and Bcl-xL from primary cultures were sorted based on GFP expression, and equal numbers of cells were re-stimulated with anti-CD3 plus anti-CD28 antibodies. Greater numbers of cells were recovered over time from Tregs derived from expression of both FoxP3 and Bcl-xL as compared with cells transduced with FoxP3 alone (Figure 3a). To evaluate Treg function, Tregs isolated from CD4^+ ^T cells expressing both FoxP3 and Bcl-xL or FoxP3 alone were co-cultured with naive CD4^+ ^T cells (Tregs/T cells = 1:1) in the presence of anti-CD3 and anti-CD28 antibodies for various periods of time. The results show that production of IL-2 and IFN-γ over the 48-hour period was significantly decreased in the presence of Tregs derived from expression of FoxP3 alone or FoxP3 plus Bcl-xL (Figure 3b). However, the proliferation in CD4^+ ^GFP^- ^T cells was significantly suppressed by Tregs co-expressing FoxP3 and Bcl-xL as compared with cells expressing FoxP3 alone on day 4 but not day 2 (Figure 3c, d). These data indicate that the transduction of both FoxP3 and Bcl-xL sustains suppressive activity of CD4^+ ^T cell-derived Tregs by promoting survival advantage.

### FoxP3 and Bcl-xL sustain survival of Tregs *in vivo*

**Figure 4:**
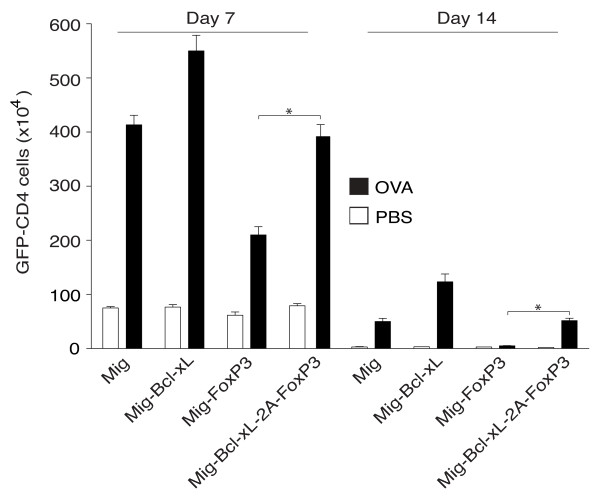
**Retrovirus-mediated transduction of FoxP3 and Bcl-xL sustains survival of regulatory T cells *in vivo***. Naive CD4^+ ^T cells from OT-II T-cell receptor transgenic mice were stimulated with peptide/antigen-presenting cells. On day 2/3, T cells were transduced with retroviral vectors expressing green fluorescent protein (GFP), GFP with Bcl-xL, GFP with FoxP3, or GFP with Bcl-xL and FoxP3. On day 6 of primary culture, 2 × 10^6 ^GFP^+ ^CD4^+ ^T cells were sorted and adoptively transferred into naive recipient mice that were subsequently challenged intraperitoneally with whole ovalbumin (OVA) protein (100 μg) in phosphate-buffered saline (PBS) (filled bars) or with PBS alone (open bars). On days 7 and 14, GFP^+ ^CD4^+ ^T cells were enumerated from pooled lymph nodes and spleen. Data are mean number of GFP^+ ^CD4^+ ^± standard error of the mean from four individual mice and representative of three independent experiments (*P <0.05, Student unpaired *t *test).

We reported previously that induced expression of Bcl-xL significantly sustained T-cell survival [15,22]. In the present study, we examined whether co-expression of Bcl-xL with FoxP3 is capable of increasing the persistence of Tregs *in vivo*. CD4^+ ^T cells from OT-II TCR transgenic mice were induced to differentiate into Tregs by transduction of FoxP3 alone or FoxP3 and Bcl-xL. On day 6 post-transduction, GFP^+ ^Tregs were sorted and adoptively transferred into syngeneic recipient mice. These mice were subsequently challenged with OVA protein. We found that the effect of Bcl-xL and FoxP3 was cooperative and long-lasting as we measured greater numbers of Tregs expressing both genes than cells derived by FoxP3 transduction alone on days 7 and 14 after Ag challenge (Figure 4). Thus, Tregs were maintained above background levels beyond the time of normal T-cell contraction (Figure 4). Overall, these data strongly support the conclusion that the combined activities of FoxP3 and Bcl-xL promote both the differentiation and long-term survival of Tregs.

### Adoptive cell transfer of FoxP3 plus Bcl-xL-transduced Tregs prevents the development of collagen-induced arthritis

**Figure 5:**
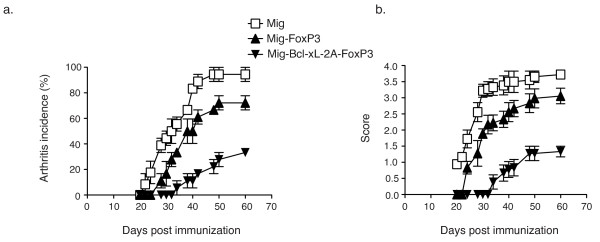
**Adoptive cell transfer of FoxP3- and Bcl-xL-transduced regulatory T cells suppresses collagen-induced arthritis (CIA)**. Naive CD4^+ ^T cells from DBA/1J mice were stimulated with anti-CD3 plus anti-CD28 antibodies. On days 2 and 3, the cells were transduced with retroviral constructs: vector (Mig), FoxP3 (Mig-FoxP3), or FoxP3 with Bcl-xL (Mig-Bcl-xL-2A-FoxP3). On day 6, green fluorescent protein-positive (GFP^+^) T cells were sorted and prepared for adoptive cell transfer. CIA was induced in male DBA/1J mice (>4 months old) by one (day 0) intradermal immunization in the base of the tail with 100 μg of bovine type II collagen in complete Freund's adjuvant, containing 5 mg/mL killed *Mycobacterium tuberculosis *(H37Ra). On day 15 after the immunization, the mice received transduced GFP^+ ^cells (2.5 × 10^6 ^per mouse, six mice per group). In the following days, the arthritis incidence **(a) **and clinical score **(b) **were evaluated by examining the paws and using a 4-point scale: 0, normal paw; 1, minimal swelling or redness; 2, redness and swelling involving the entire forepaw; 3, redness and swelling involving the entire limp; 4, joint deformity or ankylosis or both. Values are the mean ± standard error of the mean of data obtained in three experiments, and in each experiment, six mice per group were used. Summaries of the incidences and the mouse arthritis scores of the three experiments are listed in Additional files 1 and 2.

To demonstrate that the gene transduction of FoxP3 and Bcl-xL sustains the Treg response in a physiologically and clinically relevant setting, GFP-sorted Tregs derived from DBA/1J mice were adoptively transferred into male DBA/1J mice (>4 months of age). Fifteen days prior to adoptive transfer (day 0), CIA was induced in the recipient DBA/1J mice by one intradermal immunization in the base of the tail with 100 μg of CII in CFA, containing 5 mg/mL killed *Mycobacterium tuberculosis *(H37Ra). Arthritis incidence (Figure 5a) and clinical score (Figure 5b) were assessed in the paws from three independent experiments (Additional files 1 and 2). Mice receiving FoxP3-plus-Bcl-xL-transduced Tregs had a significantly decreased incidence of arthritis compared with mice receiving Tregs transduced with FoxP3 alone (32% versus 70% on day 60; Figure 5a). Most importantly, mice receiving FoxP3-plus-Bcl-xL-transduced Tregs had a lower clinical score than those receiving Tregs transduced with FoxP3 alone (a score of 1.2 versus 2.8 on day 60; Figure 5b).

**Figure 6:**
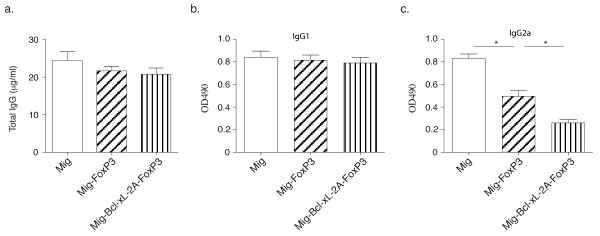
**Adoptive cell transfer of FoxP3- and Bcl-xL-transduced regulatory T cells decreases arthritis-specific antibody production**. The treatment of adoptive cell transfer for collagen-induced arthritis is described in the legend of Figure 5. On day 60 of immunization, sera were separated from all samples and the levels of serum total IgG and anti-bovine type II collagen antibodies were determined by enzyme-linked immunosorbent assay. **(a) **Total IgG. **(b) **IgG1. **(c) **IgG2a. Values are the mean ± standard error of the mean (n = 6). Data are representative of two similar experiments (*P <0.05, Student unpaired *t *test). OD, optical density.

**Figure 7:**
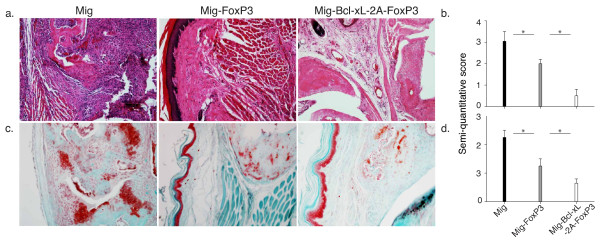
**Adoptive cell transfer of Bcl-xL- and FoxP3-transduced regulatory T cells reduces paw inflammation**. The protocol of adoptive cell transfer for collagen-induced arthritis is described in the legend of Figure 5. On day 60 of immunization, hind foot paws were amputated, fixed, and decalcified. The tissues were embedded in paraffin, sectioned, and stained. **(a) **Hematoxylin and eosin (HE) staining. **(b) **HE semiquantitative score. **(c) **Safranin O-fast green staining. **(d) **Safranin O-fast green semiquantitative score. Data are representative of two similar experiments (*P <0.05, Student unpaired *t *test).

We also examined whether disease severity correlates with antibody production and histological observations of the joints. We found that mice receiving FoxP3-plus-Bcl-xL-transduced Tregs had similar levels of total IgG and anti-CII IgG1 in the serum as mice that received Tregs transduced with FoxP3 alone (Figure 6a, b). However, anti-CII IgG2a was significantly decreased following adoptive transfer of FoxP3-plus-Bcl-xL-transduced Tregs (Figure 6c). These results are similar to observations reported in a recent study that describes decreased levels of IgG2a following adoptive transfer of Tregs [23]. Because anti-CII IgG2a was the major component of anti-CII IgG on day 60, total anti-CII IgG in this case was significantly reduced by the treatment of adoptive transfer of FoxP3-plus-Bcl-xL-transduced Tregs. These results are similar to observations previously reported [24,25]. The preventive effects of Treg transfer on CIA were also confirmed by histological analyses. In the control mice, there were significant signs of arthritic pathology as indicted by destruction of cartilage with fibrosis and leukocyte infiltration around bones and in the joint space on day 60 (Figure 7a). In contrast, the treatment of adoptive cell transfer of Tregs resulted in a marked suppression of these symptoms that are characteristic of CIA. In addition, there were only a few foci of leukocyte infiltration and less destruction of bones and joints in mice that received FoxP3-plus-Bcl-xL-transduced Tregs (Figure 7). Collectively, these findings show that the expression of FoxP3 and Bcl-xL by retrovirus-mediated transduction can cooperatively promote the persistence of Tregs and that these Tregs can effectively prevent the development of CIA.

## Discussion

Signals from TCR or co-stimulatory molecules such as CD28, 4-1BB, and ICOS or both have been shown to promote expression of Bcl-xL, which controls T-cell survival, and maintain expression of FoxP3, which sustains suppressive activities of Tregs [10,12,26-30]. In addition, naive CD4^+ ^T cells have the ability to differentiate into Tregs by gene transduction of FoxP3 [2-4], and FoxP3 transgenic mice are resistant to CIA via reduced proliferation of activated T cells [31]. Moreover, it has been shown that adoptive cell transfer of Tregs can suppress arthritis [24,32]. Therefore, we tested the hypothesis that co-transduction of CD4^+ ^T cells with both FoxP3 and Bcl-xL will generate highly reactive Tregs that can be used to prevent autoimmune disease. In this study, we show that (a) co-expression of FoxP3 and Bcl-xL is able to contribute in a cooperative manner to augment the accumulation and persistence of Tregs and (b) these Tregs efficiently prevent the onset of CIA *in vivo*.

To induce the simultaneous and equal expression of two exogenous proteins in CD4^+ ^T cells, we used the picornavirus self-cleaving T2A sequence to link genes encoding FoxP3 and Bcl-xL. The 2A-like sequences are small (54 base pairs [bp] in T2A), making multiple cistronic constructs ideal for use in size-restricted viral and non-viral vectors and promoting equal and coupled expression of several genes (total size of less than 4,000 bp) [33,34], as we have documented here. We previously reported that cooperation between molecular targets of co-stimulation promotes T-cell persistence and tumor regression [18]. In that study, also using the picornavirus 2A sequence, we linked Bcl-xL and survivin in a retroviral vector that permitted their equal expression in primary T cells. Our results showed that, by using this 2A sequence, Bcl-xL and survivin were concomitantly expressed in primary CD8^+ ^T cells after retrovirus-mediated transduction. Furthermore, Bcl-xL and survivin cooperated to sustain T-cell division and survival over time, and hence we concluded that they coordinately regulate the extent of clonal expansion of primary effector and memory effector T cells. In the present study, using the 2A sequence and a similar approach, we found that FoxP3 and Bcl-xL can also cooperatively promote differentiation and persistence of Tregs, resulting in the prevention of arthritis development.

Tregs develop in the thymus and are identified by their high levels of CD4, CD25, and FoxP3 and also their low expression of CD127 [35,36]. Expression of FoxP3 is required for Treg development and appears to control cell fate [19]. In the peripheral lymphoid organs, the large majority of FoxP3-expressing Tregs are found within the major histocompatibility complex (MHC) class II-restricted CD4-expressing (CD4^+^) helper T-cell population and express high levels of the IL-2 receptor alpha chain (CD25). In addition to the FoxP3-expressing CD4^+^CD25^+ ^Tregs, there appears to be a minor population of MHC class I-restricted CD8^+ ^FoxP3-expressing Tregs. Because CD25 is also expressed on activated T cells, the regulatory T-cell population is more accurately defined by FoxP3 expression. Typically, high levels of CTLA-4 and glucocorticoid-induced TNF receptor (GITR) are also expressed on Tregs [37-39], but the functional significance of this expression remains to be defined. There is great interest in identifying cell surface markers that are uniquely and specifically expressed on all FoxP3-expressing Tregs. However, to date, no such molecules have been identified.

FoxP3-expressing Tregs are crucial mediators of peripheral tolerance-suppressing autoimmune responses. Induced expression of FoxP3 confers a Treg phenotype to conventional T cells, allowing these Tregs to be used therapeutically for the prevention of autoimmunity and transplant rejection. Several groups have investigated the potential use of Tregs in the treatment of arthritis and their results indicate that adoptive cell transfer of Tregs can be used therapeutically in arthritis, such as CIA [24,32,40,41]. A single transfer of CD4^+^CD25^+ ^T cells from spleens markedly slowed CIA progression, which could not be attributed to losses of systemic type II collagen-specific T- and B-cell responses [24]. Similarly, adoptive cell transfer of CD4^+^CD25^+ ^T cells from spleens and lymph nodes into immunized mice at the time of induction of CIA decreased the severity of disease but was not able to cure established arthritis [32]. Moreover, adoptive cell transfer of FoxP3-transduced Tregs significantly suppressed the progression of established CIA [40,41]. It has been suggested that the timing of Treg transfer should precede immunization in order to obtain better results since late-stage aggressive arthritis is more resistant to Treg transfer [32,41].

We considered that resistance to Treg transfer is due to Treg apoptosis. In some disease conditions, such as cancer and arthritis, Tregs were significantly more sensitive to apoptosis than normal conventional T cells (for example, non-Tregs) [42-45]. Thus, the combination of increasing the Treg number and decreasing their sensitivity to apoptosis may benefit clinical uses of Tregs for autoimmune diseases.

Tregs are highly susceptible to apoptosis in the absence of common gamma chain (γc) cytokines (for example, IL-2, IL-4, IL-7, IL-9, IL-15, and IL-21) because they do not produce these cytokines [46,47]. In addition, Tregs are susceptible to apoptosis due to low expression of the anti-apoptotic Bcl-2 family members [48]. FoxP3 transduction by itself has a detrimental effect on expansion and survival of CD4^+ ^T cells. Our data show CD4^+ ^T cells transduced by FoxP3 alone had lower cell recovery both *in vitro *and *in vivo *in comparison with vector control-transduced cells (Figures 3 and 4). This defect was then corrected by expression of Bcl-xL. Bcl-xL is an anti-apoptotic factor that could prolong the life span of apoptosis-prone lymphocytes. We used this approach to generate therapeutically reactive Tregs with an increased life span and hence greater therapeutic potential than was achieved by Foxp3 expression alone (Figures 6 and 7). Thus, the FoxP3 and Bcl-xL-transuced Tregs that we generated in this study are more efficacious than unmanipulated naturally occurring Tregs.

In this study, we induced highly reactive Tregs from CD4^+ ^T cells by retrovirus-mediated introduction of FoxP3 and Bcl-xL. This approach promoted Treg differentiation and long-term survival, resulting in long-lasting Treg persistence. In addition, adoptive cell transfer of these highly reactive Tregs prevented the development of arthritis in a murine model. These data provide new insights toward the generation of highly reactive Tregs for adoptive immunotherapy of autoimmune disease. However, adoptive cell transfer did not cure established arthritis, which might be explained by the absence of specificity that directs the movement of Tregs to the inflamed paw [25,40,49]. Thus, generation of antigen-specific highly reactive Tregs may be a promising approach for the treatment of established autoimmune disease.

## Conclusions

The results of the present study demonstrate that FoxP3 and Bcl-xL can cooperatively promote the differentiation and persistence of Tregs, thereby resulting in prevention of arthritis. The data suggest a potential novel approach to generate highly reactive Tregs for augmenting cellular immunotherapy for autoimmune disease.

## Abbreviations

bp: base pairs; BrdU: 5-bromo-2-deoxyuridine; CFA: complete Freund's adjuvant; CIA: collagen-induced arthritis; CII: bovine type II collagen; CTLA-4: cytotoxic T-lymphocyte-associated molecule-4; ELISA: enzyme-linked immunosorbent assay; FoxP3: forkhead box p3; GFP: green fluorescent protein; HE: hematoxylin and eosin; IFNγ: interferon-gamma; IL: interleukin; MHC: major histocompatibility complex; OVA: ovalbumin; PBS: phosphate-buffered saline; TCR: T-cell receptor; TGF-β1: transforming growth factor-beta 1; Treg: regulatory T cell.

## Competing interests

The authors declare that they have no competing interests.

## Authors' contributions

RH and FL carried out all experiments. XX performed the statistical analysis. JS and YW conceived of the study, participated in its design and coordination, and helped to draft the manuscript. All authors read and approved the final manuscript.

## Supplementary Material

Additional file 1 **Summary of the arthritis incidences of the three experiments**. PDF file containing a table that lists a summary of the arthritis incidences of three experiments. Click here for file

Additional file 2 **Summary of the mouse arthritis scores of the three experiments**. PDF file containing a table that lists a summary of the mouse arthritis scores of three experiments. Click here for file
