# Supplementary material for: FoxP3 and Bcl-xL cooperatively promote regulatory T cell persistence and prevention of arthritis development
Source: Arthritis Res Ther. 2010 Apr 12;12(2):R66. doi: 10.1186/ar2983 (PMC2888221; doi:10.1186/ar2983)
Supplement: Additional file 1 — Summary of the arthritis incidences of the three experiments. PDF file containing a table that lists a summary of the arthritis incidences of three experiments. [file ar2983-S1.PDF]

Table 1: Summary of the arthritis incidences of the three experiments

Days	Arthritis Incidence (%)		
(post immunization)	Mig	Mig-FoxP3	Mig-Bcl-xL-2A-FoxP3
10	0, 0, 0	0, 0, 0	0, 0, 0
20	0, 0, 0	0, 0, 0	0, 0, 0
22	16.7, 0, 16.7	0, 0, 0	0, 0, 0
24	33.3, 0, 33,3	0, 0, 0	0, 0, 0
28	50.0, 33.3, 33.3	16.7, 0, 16.7	0, 0, 0
30	50.0, 33.3, 50.0	33.3, 0, 16.7	0, 0, 0
32	66.6, 33.3, 50.0	33.3, 16.7,33.3	0, 0, 0
34	66.6, 50.0, 50.0	33.3, 33.3,33.3	16.7, 0, 0
38	66.6, 66.6, 66.6	50.0, 33.3,66.6	16.7, 16.7, 0
40	83.3, 83.3, 83.3	50.0, 33.3,66.6	16.7, 16.7, 0
42	83.3, 100.0, 83.3	66.6, 50.0,66.6	16.7, 16.7, 16.7
48	100,0, 100.0, 83.3	66.6, 66.6,66.6	33.3, 16,7, 16.7
50	100,0, 100.0, 83.3	83.3, 66.6,66.6	33.3, 16,7, 33.3
60	100,0, 100.0, 83.3	83.3, 66.6,66.6	33.3, 16,7, 50.0
