# Supplementary material for: FoxP3 and Bcl-xL cooperatively promote regulatory T cell persistence and prevention of arthritis development
Source: Arthritis Res Ther. 2010 Apr 12;12(2):R66. doi: 10.1186/ar2983 (PMC2888221; doi:10.1186/ar2983)
Supplement: Additional file 2 — Summary of the mouse arthritis scores of the three experiments. PDF file containing a table that lists a summary of the mouse arthritis scores of three experiments. [file ar2983-S2.PDF]

Table 2: Summary of the mouse arthritis scores of the three experiments

Days	Score		
(post immunization)	Mig	Mig-FoxP3	Mig-Bcl-xL-2A-FoxP3
10	0, 0, 0	0, 0, 0	0, 0, 0
20	1.00, 1.00, 0.83	0, 0, 0	0, 0, 0
22	1.33, 1.00, 1.17	0, 0, 0	0, 0, 0
24	2.00, 1.17, 2.00	0.50, 1.17, 0.83	0, 0, 0
28	3.00, 2.00, 2.67	0.50, 1.50, 1.83	0, 0, 0
30	3.50, 2.83, 3.33	2.17, 1.67, 1.83	0, 0, 0
32	3.50, 2.83, 3.50	2.67, 1.67, 2.17	0, 0, 0
34	3.67, 2.83, 3.50	2.67, 1.83, 2.17	0.83, 0.17, 0.17
38	3.83, 2.83, 3.50	2.83, 2.00, 2.17	1.00, 0.17, 0.17
40	3.83, 2.83, 3.83	3.00, 2.00, 2.67	1.17, 0.33, 0.83
42	3.83, 2.83, 3.83	3.00, 2.17, 2.83	1.17, 0.33, 1.00
48	3.83, 3.00, 3.83	3.30, 2.30, 2.90	1.50, 0.83, 1.50
50	3.83, 3.30, 3.83	3.35, 2.40, 3.20	1.50, 0.83, 1.50
60	3.83, 3.50, 3.83	3.50, 2.67, 3.00	1.50, 1.00, 1.50
